# Factors Distinguishing Proximal and Distal Internal Carotid Artery Occlusions in Patients with Acute Ischemic Stroke

**DOI:** 10.3390/diagnostics12020494

**Published:** 2022-02-14

**Authors:** Sang Woo Ha, Chan-Hyuk Lee, Hak Sung Kim, Eung Koo Yeon, Seung Jae Lee, Byoung-Soo Shin, Hyun Goo Kang

**Affiliations:** 1 Department of Neurosurgery, Chosun University Medical School, Gwangju 61453, Korea; neuroha@gmail.com (S.W.H.); loverei82@naver.com (H.S.K.); 2 Department of Neurology, Jeonbuk National University Hospital, Jeonju 54907, Korea; bluewave0210@gmail.com (C.-H.L.); sbsoo@jbnu.ac.kr (B.-S.S.); 3 Department of Neurology, Research Institute of Clinical Medicine of Jeonbuk National University-Biomedical Research Institute of Jeonbuk National University Hospital, Jeonju 54907, Korea; 4 Department of Radiology, Seoul National University Hospital, Seoul 03080, Korea; doorman38@naver.com; 5 Department of Chemistry, Institute for Molecular Biology and Genetics, Jeonbuk National University, Jeonju 54896, Korea; slee026@jbnu.ac.kr

**Keywords:** acute ischemic stroke, computed tomography angiography, digital subtraction angiography, internal carotid artery occlusion, thrombectomy

## Abstract

Acute internal carotid artery (ICA) occlusions cause extensive brain ischemia. Accurate determination of the occlusion site facilitates rapid revascularization interventions and improves prognosis. However, proximal ICA occlusions, as determined with computed tomography (CT) angiography, often are located more distally. Therefore, we assessed clinical and imaging factors associated with the accurate determination of occlusion sites. In this observational study, we evaluated 102 patients who presented acute ischemic stroke symptoms and had a CT angiography within 6 h, showing proximal ICA occlusion. The participants were divided into two groups, depending on whether there was correspondence between digital subtraction angiography and CT angiography regarding the occlusion location. Proximal occlusions were, accordingly, categorized as “true” (correspondence) or “false” (no correspondence; distal). Demographic, clinical, and imaging features were analyzed. Multivariate regression analysis was performed to identify factors predicting the correspondence between actual ICA occlusion sites and those detected by CT angiography. The shape (Odds ratios, OR = 646.584; Confidence interval, CI = 21.703–19263.187; *p* < 0.001) and the length (OR = 0.696; CI = 0.535–0.904; *p* = 0.007) of the ICA occlusion and atrial fibrillation (OR = 0.024; CI = 0.002–0.340; *p* = 0.006) were significant factors. The cut-off length of ICA stump at 6.2 mm, the sensitivity was 71%, and the specificity was 70% (area under the ROC curve = 0.767).

## 1. Introduction

The internal carotid artery (ICA) supplies blood to approximately 70% of the brain parenchyma. Therefore, ICA occlusions often result in ischemic strokes affecting an extensive brain volume, and the prognosis of patients with such occlusions is often poor [1]. In most cases, an ischemic stroke is caused by atherosclerosis in a major artery or cardioembolism [2,3].

When a patient suspected to have stroke is hospitalized, non-enhanced brain computed tomography (CT) is performed initially to rule out intracranial hemorrhage. If the results are negative, CT angiography is then performed to examine the major cerebral arteries. Compared with magnetic resonance angiography, CT angiography allows cerebrovascular examination in a timelier fashion [4]. The ICA does not have other major branches until the anterior cerebral artery, except the ophthalmic, anterior choroidal, and posterior communicating arteries. Hence, based on CT angiography, an ICA occlusion may be defined as proximal regardless of the actual occlusion site [5,6,7]. For patients with suspected ICA occlusion, determining the actual occlusion site before intra-arterial (IA) thrombectomy is paramount because the required surgical devices and catheters vary depending on the artery diameter at the occlusion. If the occlusion site can be accurately localized, the most appropriate surgical devices can be prepared in advance. Moreover, the interventionist may reach the occlusion site more quickly, possibly improving prognosis [8].

In this study, we examined the factors indicating the correct determination of the occlusion site in patients with acute ischemic stroke showing as a proximal ICA occlusion based on CT angiography.

## 2. Materials and Methods

### 2.1. Study Population

This observational study reviewed the data of patients aged ≥19 years who presented to a single center between January 2016 and October 2019 (46 months). The patients underwent CT angiography within 6 h of neurological symptom onset. In total, 111 patients who had been diagnosed with proximal ICA occlusion causing the neurological symptoms were enrolled. Nine patients with common carotid artery (CCA) occlusion or spontaneous recanalization of the occluded ICA after an intravenous thrombolytic agent (tissue plasminogen activator, tPA) were excluded. Hence, 102 patients were finally evaluated.

This study was approved by the Institutional Review Board (approval number: 2020-03-029). All procedures were performed according to the ethical standards of the institutional and national research committees and the Declaration of Helsinki. The need to obtain informed consent was waived due to the nature of the study.

### 2.2. Acute Ischemic Stroke Management

Patients without acute intracranial hemorrhage were administered tPA, according to the 2019 American Heart Association/American Stroke Association (AHA/ASA) guidelines. If a major cerebral artery of an admitted patient was confirmed to be occluded within 6 h of neurological symptom onset, an IA thrombectomy was considered. If a patient had a National Institutes of Health Stroke Scale (NIHSS) score ≥6 and an Alberta Stroke Program Early CT Score (ASPECTS) ≥6, IA thrombectomy was performed. An interventionist inserted a guidewire into the right femoral artery under ultrasonographic guidance; then, a vascular sheath was introduced into the blood vessel. Afterward, a catheter (Fubuki Neurovascular Guide Catheter, Asahi Intecc CO LTD., Aichi, Japan) was inserted into the vascular sheath and advanced to the CCA. The patency of the vessel and its collaterals was confirmed by injecting a contrast medium after the removal of the guidewire. After the occlusion was confirmed, mechanical thrombectomy was performed using aspiration (ACE 68 Reperfusion Catheter, Penumbra, Inc., Alameda, CA, USA), a stentriever (Solitaire FR 2, Medtronic, Sunnyvale, CA, USA), or both, depending on the vessel diameter. We only enrolled patients with proximal ICA occlusions visible on CT angiographs.

### 2.3. Data Acquisition and Classification Criteria

**Figure 1:**
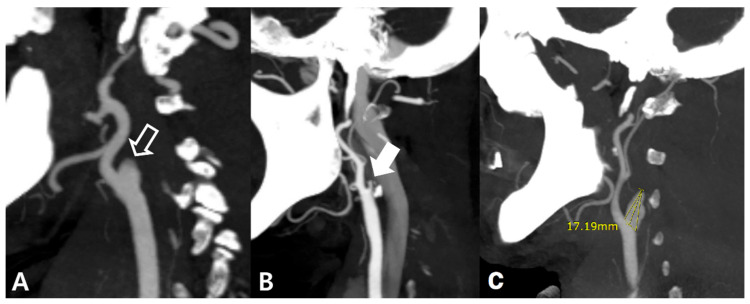
Images of the proximal internal carotid artery occlusion on computed tomography angiography ((**A**): blunt shape, empty arrow; (**B**): not-blunt shape, white arrow; (**C**): length of the ICA stump measurement).

We classified those who showed occlusion at proximal ICA in CT angiography, when digital subtraction angiography (DSA) was conducted for this patient, if occlusion was seen in the same area, it was defined as true proximal ICA occlusion and those who showed distal ICA occlusion in DSA, unlike CT angiography, as false proximal ICA occlusion (Supplementary Figure S1). All electronic medical records and imaging data were analyzed. The patients were also subdivided into the blunt and not-blunt groups based on the occlusion site contour: a closed semi-lunar shape with a gentle curve was classified as a blunt contour, whereas other shapes as not-blunt contours (Figure 1A,B).

A neurosurgeon and a neurologist evaluated the shape of the occlusion. The opinion of another expert (a radiologist, E.K.Y.) was sought when a consensus could not be reached. Calcification was identified in the presence of an area with density >500 Hounsfield units and size >1 mm^2^ within 3 cm of the proximal ICA origin on CT angiography [9] and a carotid calcium score ≥100. The length of each ICA stump was manually measured using the maximum intensity projection on CT angiography (Figure 1C). There was a perfusion mismatch if the area with a Tmax ≥6 s was >20% greater than the area with a cerebral blood flow <30% on perfusion CT [10].

In-situ stenosis was defined when there was more than moderate (>50%) chronic stenosis of proximal ICA even after mechanical thrombectomy was performed.

### 2.4. Statistical Analyses

The Student’s *t*-test was used to analyze the continuous variables and the Pearson’s chi-squared test for the categorical variables. Statistical significance was set at *p* ≤ 0.05. Logistic regression analysis was performed to identify the factors predicting the correctly determined proximal ICA occlusions. First, we analyzed all each factors by using univariate logistic regression analysis. Then, only the factors with *p* ≤ 0.05 were regathered for multivariate logistic regression analysis. The results of the multivariate logistic regression analysis are presented as *p*-values, adjusted odds ratios (aORs), and 95% confidence intervals (CIs). Receiver operating characteristic (ROC) curves were plotted to determine the sensitivity and specificity of carotid CT angiography for determining the length of the ICA stump. All statistical analyses were performed using SPSS 25.0 for Windows (IBM Corp., Armonk, NY, USA).

## 3. Results

### 3.1. General Characteristics

This study analyzed 102 patients (false occlusion group, *n* = 62; true occlusion group, *n* = 40) with a mean age of 74.6 ± 11.3 years (false occlusion group = 76.4 ± 11.0; true occlusion group = 71.9 ± 11.5). The proportion of male patients was higher in the true occlusion group than in the false occlusion group (80% vs. 41.9%; *p* < 0.001), whereas the proportion of patients with atrial fibrillation (59.7% vs. 7.5%; *p* < 0.001) and a history of ischemic stroke (24.2% vs. 7.5%, *p* < 0.035) were higher in the false occlusion group than in the true occlusion group (Table 1).

### 3.2. Ischemic Stroke Related Characteristics

The severity of neurological symptoms on admission did not significantly differ in the two groups; however, the modified Rankin Scale scores at the time of discharge were significantly lower in the true occlusion group than in the false occlusion group. The two groups did not show significant differences in terms of the 3-month modified Rankin Scale scores. Based on the brain CT findings, the true occlusion group showed a higher perfusion mismatch rate than the false occlusion group (97.4% vs. 80.6%, *p* = 0.015; Table 2).

On CT angiography, the proportion of blunt-shaped occlusions was higher in false group than the opposite (false occlusion group: 95.2%; true occlusion group: 20%; *p* < 0.001). Conversely, calcifications and in situ stenosis were more in the true group (false occlusion group: 53.2%, true occlusion group: 75.0%, *p* = 0.027; false occlusion group: 22.6%, true occlusion group: 95.0%; *p* < 0.001, respectively). These data are shown in Table 2.

### 3.3. Associated Factors of Proximal ICA Occlusion

**Figure 2:**
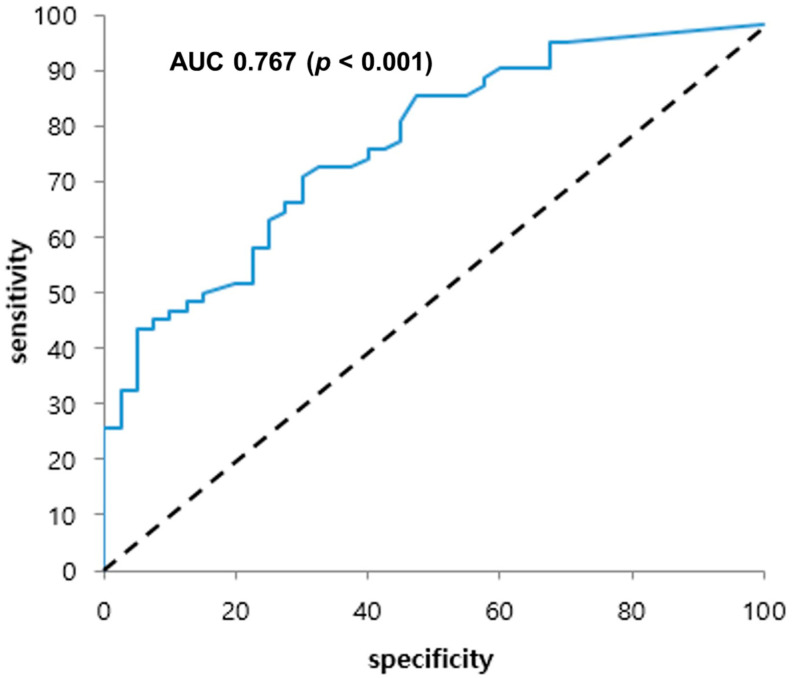
Receiver operating characteristic curve for the length of the internal carotid artery stump based on carotid computed tomography angiography. The probability of an internal carotid stump under 6.2 mm of being a correctly localized proximal occlusion was analyzed; sensitivity of 71% and a specificity of 70% were noted. Area under the receiver operating characteristic curve: 0.767; *p* < 0.001; 95% confidence interval: 0.676–0.858. AUC: area under the curve.

Multivariate logistic regression analysis was performed to identify the factors the localization of the true occlusion site. The non-blunt shape (OR = 646.584; CI = 21.703–19263.187; *p* < 0.001) and the length (OR = 0.696; CI = 0.535–0.904; *p* = 0.007) of the ICA stump and atrial fibrillation (OR = 0.024; CI = 0.002–0.340; *p* = 0.006) were significant factors (Table 3). The receiver operating characteristic (ROC) curve analysis was used to determine the sensitivity and specificity for the length of the ICA stump; the length was 6.2 mm, the sensitivity was 71%, and the specificity was 70% (area under the ROC curve = 0.767; Figure 2).

## 4. Discussion

In this study, we divided acute ischemic stroke patients diagnosed with proximal ICA occlusion based on CT angiography findings into two groups, depending on the correspondence between the predicted and actual occlusion locations. Our results showed that the shape and length of the ICA stump and atrial fibrillation indicated the accuracy of the predicted occlusion location compared to the angiography findings.

Hong et al. [11] examined 49 acute ischemic stroke patients with proximal ICA occlusion on CT angiography. They divided the patients into three groups (stump, spearhead, and streak groups) according to the shape of the ICA occlusion; proximal occlusions were more frequently reported in the stump group, whereas distal occlusions were mainly reported in the spearhead and streak groups. Another study categorized the shapes of the ICA occlusion into flat, beak, and dome types and reported that the flat type was associated with proximal occlusions, whereas the beak type with distal occlusions. These results contradicted the findings of a previous study [12]. Our results showed that distal ICA occlusions have a blunt shape. When the distal ICA is completely occluded, the blood flow from the CCA bifurcation to the ICA stagnates. This dynamic hinders the laminar flow and consequently increases turbulence. We speculate that distal ICA occlusions are blunt due to this mechanism. The studies by Hong et al. [11] and Kim et al. [12] involved small samples (N = 49 and N = 66, respectively) and used different ICA occlusion shape criteria; hence, the results should be interpreted with caution. Separately, it cannot be excluded that the thrombus in the proximal segment migrated to the distal area by the catheter manipulation used for thrombectomy.

In addition, our study revealed that patients with atrial fibrillation were more likely to have distal ICA occlusions. Atrial fibrillation leads to blood stasis in the heart, causing thrombi formation. These thrombi separate from the vessel walls and travel within the blood vessels, eventually blocking the distal cerebral arteries and causing ischemic strokes. The proximal ICA is wider than the distal ICA; moreover, since cardioembolic stroke is not associated with significant carotid stenosis, the probability of an embolic occlusion within the proximal carotid is relatively low. Therefore, the distal ICA is more likely to be occluded [13].

Our study showed that a short carotid stump after the CCA bifurcation was associated with a high probability of a true proximal ICA occlusion. Atherosclerotic changes in the carotid artery mainly occur in the proximity of the carotid bifurcation [14]. When the ICA is occluded due to the progression of atherosclerotic plaques, stumps are likely to form near the bifurcation. Our results might imply that when the occlusion is atherosclerotic, the occluded stump may be relatively shorter than when due to other etiologies.

Accurately locating the carotid occlusion in patients with acute ischemic stroke can help interventionists plan an IA thrombectomy. Aspiration and stent retriever thrombectomy are first-line approaches; in recent years, a combination of these techniques has also been used. Depending on the condition of the blood vessels and the skills of the interventionist, diverse combinations of devices (e.g., femoral sheath, guiding catheter with/without balloon guiding, suction catheter, and microcatheter) are available. By selecting the most appropriate device, the invasive procedure can be adequately planned, the number of attempts minimized, and the procedural time reduced. These factors affect patient outcomes and complication occurrence. Additionally, planning the appropriate methodology can prevent excessive medical costs.

This study has some limitations. First, it was designed as an observational study. Designing a cohort study was difficult since we targeted patients with suspected acute proximal ICA occlusion. However, a prospective cohort study can be conducted for patients with atrial fibrillation or carotid artery calcifications. Second, this study analyzed relatively few patients from a single center; hence, a larger multicenter trial is needed to confirm the study findings. Third, individual factors during data collection, such as variable interpretation of the ICA occlusion shape, may have affected the outcomes. However, multiple researchers participated in the classification to minimize this possibility. Furthermore, the results of the multivariate regression analysis regarding the shape of the ICA occlusions were more significant than expected, possibly because only three cases were considered “not blunt” in the false occlusion group.

## 5. Conclusions

The shape and length of the ICA stump and atrial fibrillation were predictors of the actual location of ICA occlusions, classified as proximal on CT angiography. The ICA is the main vessel that supplies blood to the anterior circulation, and its occlusion can damage most of the supratentorial areas. Rapid thrombi removal with IA thrombectomy can minimize ischemic damage to the brain. If the ICA occlusion site can be accurately predicted, treatment preparation and interventional procedures can be optimized. Accurate planning will shorten the procedural duration, minimize side effects, and improve the prognosis of patients with ICA occlusions.

## Figures and Tables

**Table 1:** Baseline characteristics of the study population.

Variables	Carotid Occlusion		*p*-Value
	False (*n* = 62)	True (*n* = 40)	
**Demographics**			
Age	76.4 ± 11.0	71.9 ± 11.5	0.052
Male, *n* (%)	26 (41.9)	32 (80)	<0.001
**Conventional risk factors, *n* (%)**			
Hypertension	42 (67.7)	21 (52.5)	0.122
Diabetes mellitus	14 (22.6)	9 (22.5)	0.992
Hyperlipidemia	6 (9.7)	2 (5.0)	0.476
Atrial fibrillation	37 (59.7)	3 (7.5)	<0.001
Previous stroke	15 (24.2)	3 (7.5)	0.035
Previous CAD	6 (9.7)	3 (7.5)	1.000
Smoking	9 (14.5)	9 (22.5)	0.302
Alcohol consumption	10 (16.1)	10 (25.0)	0.271
**Neurological Severity**			
Previous mRS	0 [0–0]	0 [0–0]	0.070
Initial NIHSS	15 [12–18]	13 [9–17.75]	0.300
Discharge mRS	4 [3–5]	4 [1.25–4.75]	0.023
Discharge NIHSS	12.5 [5–20.25]	10 [4–14.75]	0.177
3-month mRS ^1^	4 [1.5–6]	3 [0.75–4]	0.076

^1^ False: *n* = 32, True: *n* = 26. Values are presented as the number (%) of patients or mean ± standard deviation/median [interquartiles]. CAD: coronary artery disease; mRS: modified Rankin Scale; NIHSS: National Institutes of Health Stroke Scale.

**Table 2:** Characteristics of the study population related to acute ischemic stroke management.

Variables	Proximal ICA Occlusion		*p*-Value
	False (*n* = 62)	True (*n* = 40)	
**Brain CT**			
ASPECT score	7 [5–9]	8 [7–9]	0.047
Perfusion mismatch ^1^	50 (80.6)	38 (97.4)	0.015
Intravenous tPA	27 (43.5)	15 (37.5)	0.545
**State of carotid ICA**			
Blunt	59 (95.2)	8 (20.0)	<0.001
In-situ stenosis	14 (22.6)	38 (95.0)	<0.001
Calcification	33 (53.2)	30 (75.0)	0.027
Length of ICA stump (mm)	12.7 ± 9.1	5.1 ± 4.5	<0.001
**Occlusion site**			
Proximal ICA	0 (0.0)	17 (42.5)	<0.001
Distal ICA	42 (67.7)	0 (0.0)	
M1 (T-occlusion)	20 (32.3)	0 (0.0)	
Proximal ICA with M1	0 (0.0)	23 (57.5)	
**IA Thrombectomy Results**			
TICI			
I	6 (9.7)	1 (2.5)	0.356
IIa	1 (1.6)	2 (5.0)	
IIb	5 (8.1)	2 (5.0)	
III	50 (80.6)	35 (87.5)	
**Hemorrhagic complication**			
No	30 (48.4)	16 (40.0)	0.473
HI	19 (30.6)	17 (42.5)	
PH	13 (21.0)	7 (17.5)	

^1^ False: *n* = 62, True: *n* = 39. Values are presented as the number (%) of patients or mean ± standard deviation/median [interquartiles]. ICA: internal carotid artery; CT: computed tomography; ASPECTS: Alberta stroke program early CT score; tPA: tissue plasminogen activator; IA: intra-arterial; TICI: thrombolysis in cerebral infarction; HI: hemorrhagic infarction; PH: parenchymal hematoma.

**Table 3:** Logistic regression analysis of true proximal ICA occlusions.

Variables	Univariate Analysis		Multivariate Analysis	
	Crude OR (95% CI)	*p*-Value	Adjusted OR (95% CI)	*p*-Value
ICA stump not blunt	78.667 (19.498–317.395)	<0.001	646.584 (21.703–19263.187)	<0.001
Length of ICA stump	0.85 (0.783–0.924)	<0.001	0.696 (0.535–0.904)	0.007
Calcification	2.636 (1.102–6.308)	0.029	1.255 (0.202–7.800)	0.807
Atrial fibrillation	0.055 (0.015–0.197)	<0.001	0.024 (0.002–0.340)	0.006

*p*-values indicate the significance of the multivariable logistic regression results. Variables with *p* < 0.1 in the univariate analysis were entered into the multivariate analysis model. ICA: internal carotid artery; OR: odds ratio; CI: confidence interval.

## Data Availability

Data are available on reasonable request.

## Supplementary Materials

The following supporting information can be downloaded at: https://www.mdpi.com/article/10.3390/diagnostics12020494/s1, Figure S1: Image differences between true and false proximal internal carotid artery (ICA) occlusion.
