# Supplementary material for: Factors Distinguishing Proximal and Distal Internal Carotid Artery Occlusions in Patients with Acute Ischemic Stroke
Source: Diagnostics (Basel). 2022 Feb 14;12(2):494. doi: 10.3390/diagnostics12020494 (PMC8871289; doi:10.3390/diagnostics12020494)
Supplement: Supplementary file 1 [file diagnostics-12-00494-s001.zip › diagnostics-1519635-supplementary.pdf]

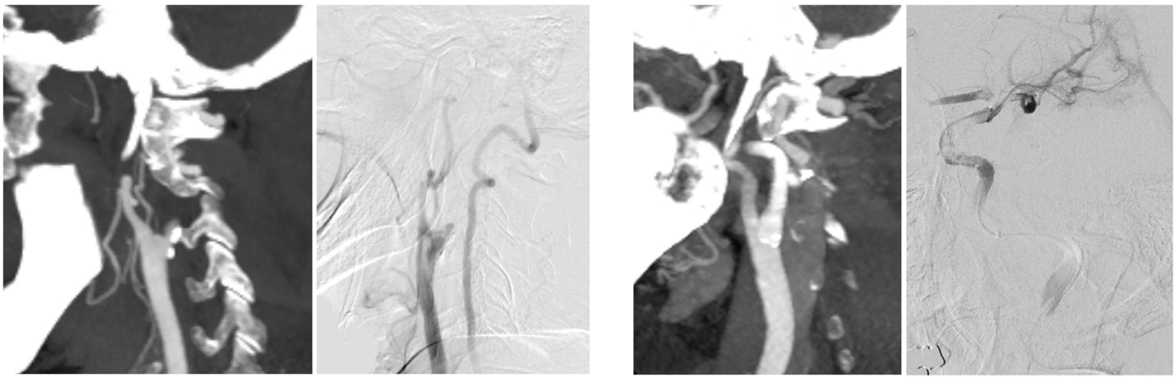

<True> <False>

Figure S1. Image differences between true and false proximal internal carotid artery (ICA) occlusion. True proximal ICA occlusion means that proximal ICA occlusion is confirmed at the same location in both brain computed tomography (CT) angiography and digital subtraction angiography (DSA). False proximal ICA occlusion means that proximal ICA occlusion is confirmed on CT agniography, whereas DSA showed distal ICA occlusion.
